# Endiandric Acid Derivatives and Other Constituents of Plants from the Genera *Beilschmiedia* and *Endiandra* (Lauraceae)

**DOI:** 10.3390/biom5020910

**Published:** 2015-05-14

**Authors:** Bruno Ndjakou Lenta, Jean Rodolphe Chouna, Pepin Alango Nkeng-Efouet, Norbert Sewald

**Affiliations:** 1 Department of Chemistry, Higher Teacher Training College, University of Yaoundé 1, P.O. Box 47, Yaoundé, Cameroon; 2 Organic and Bioorganic Chemistry, Chemistry Department, Bielefeld University, P.O. Box 100131, 33501 Bielefeld, Germany; E-Mail: norbert.sewald@uni-bielefeld.de; 3 Department of Chemistry, University of Dschang, P.O. Box 67, Dschang, Cameroon; E-Mails: chounajr@yahoo.fr (J.R.C.); pnfalango@yahoo.fr (P.A.N.-E.)

**Keywords:** Lauraceae, *Beilschmiedia*, *Endiandra*, chemical constituents, endiandric acid, alkaloids, biological activities

## Abstract

Plants of the Lauraceae family are widely used in traditional medicine and are sources of various classes of secondary metabolites. Two genera of this family, *Beilschmiedia* and *Endiandra*, have been the subject of numerous investigations over the past decades because of their application in traditional medicine. They are the only source of bioactive endiandric acid derivatives. Noteworthy is that their biosynthesis contains two consecutive non-enzymatic electrocyclic reactions. Several interesting biological activities for this specific class of secondary metabolites and other constituents of the two genera have been reported, including antimicrobial, enzymes inhibitory and cytotoxic properties. This review compiles information on the structures of the compounds described between January 1960 and March 2015, their biological activities and information on endiandric acid biosynthesis, with 104 references being cited.

## 1. Introduction

The family Lauraceae is one of the most important groups of Angiosperms and consists of 55 genera and over 2000 species [1,2,3,4,5]. *Beilschmiedia* is one of the largest pantropical genera in the Lauraceae, comprising about 250 species [6,7,8]. Most of its species grow in tropical climates, but few of them are native to temperate regions, and they are widespread in tropical Asia, Africa, Madagascar, Australia, New Zealand, North America, Central America, the Caribbean, and South America [6,7,8]. Regarding *Endiandra*, there are about 125 species found throughout the tropical regions, including 10 species in Malaysia [3,9,10,11]. Very little information is available on the medicinal use of plants of the *Endiandra* genus, but some *Beilschmiedia* species have been used in the indigenous system of medicine for the treatment of various disorders such as uterine tumours, rubella, female genital infections, rheumatism, colon and digestive disorders, malaria, headache, as well as bacterial or fungal infections [12,13,14]. The fruit of some species are used as appetite stimulants and also as spices [15,16,17,18].

*Beilschmiedia* and *Endiandra* species have been known for a long time as rich sources of biologically active secondary metabolites. They have been the subject of very intensive chemical investigations by various research groups starting from the middle of the 1960s, with a large number of compounds isolated from different species. However, phytochemical investigations have been mostly conducted on 31 species of *Beilschmiedia* (*Beilschmiedia alloiophylla*, *B. anacardioides*, *B. bernesii*, *B. brevipes*, *B. chancho chancho*, *B. collina*, *B. costaricensis*, *B. cryptocaryoides*, *B. elliptica*, *B. erythrophloia*, *B. fulva*, *B. tsangii*, *B. ferruginea*, *B. kunstleri*, *B. madang*, *B. manii*, *B. miersii*, *B. obscura*, *B. obtusifolia*, *B. oligandra*, *B. oreophila* Schlechter, *B. pendula*, *B. podagrica*, *Beilschmiedia spp* (from Gabon), *B. talaranensis*, *B. tarairie*, *B. tawa*, *B. tooram*, *B. tovarensis*, *B. volckii*, and *B. zenkeri*) [12,13,15,16,17,18,19,20,21,22,23,24,25,26,27,28,29,30,31] and 11 species of *Endiandra* (*Endiandra anthropophagorum*, *E. bassaphila*, *E. baillonii*, *E. introsa*, *E. jonesii*, *E. kingiana*, *E. leptodendron*, *E. monothyra*, *E. palmerstonii*, *E. xanthocarpa*, *and E. wolfii*) [32,33,34,35,36,37,38,39,40,41,42,43]. These investigations led to the isolation and characterization of various classes of secondary metabolites, of which endiandric acid derivatives, epoxyfuranoid lignans, kingianins (compounds with unique pentacyclic skeleton), and alkaloids exhibited antibacterial, anti-inflammatory, and anticancer activities and inhibited different enzymes [17,18,19,20,21,22,23,24,25,26,27,28,29,30,31,32,33,34,35,36,37,38,39,40,41,42,43,44,45,46,47,48,49,50,51,52,53,54]. Endiandric acid derivatives, polycyclic fatty acids that possess among others also anti-asthmatic activity have been isolated only from plants of the genera *Beilschmiedia* and *Endiandra*. The biosynthesis of this class of secondary metabolites contains two consecutive non-enzymatic electrocyclic reactions [35,36,55].

## 2. Chemical Constituents

### 2.1. Endiandric Acid Derivatives from *Beilschmiedia* and *Endiandra*

Plants of the genera *Beilschmiedia* and *Endiandra* have been known for a long time as rich source of endiandric acid derivatives. They are still the only sources of this class of secondary metabolites. Endiandric acid derivatives have been found in 11 species of *Beilschmiedia* (*Beilschmiedia anacardioides*, *B. alloiophylla*, *B. cryptocaryoides*, *B. erythrophloia*, *B. ferruginea*, *B. fulva*, *B. manii*, Gaboneses *Beilschmiedia spp*, *B. oligandra*, *B. obtusifolia*, *B. tooram*, and *B. tsangii*) [17,19,20,22,23,24,44,48,52,53,54] and in 4 species of *Endiandra* (*Endiandra baillonii*, *E. introsa,* *E. jonesii*, and *E. kingiana*) [34,40]. All these compounds have some structural properties in common, such as the cyclic nature, the presence of double bonds and terminal carboxylic acid groups. They can be grouped according to the carbon skeleton layout into three groups. The first group is that of compounds characterized by a 13 carbon atom fused tetracyclic ring system containing Δ^8,9^ and Δ^4,5^ or Δ^5,6^ (**1**); the second group is that of compounds with tetracylic ring system formed with 11 carbon atoms with Δ^10,11^ (**2**) and the last group contains compounds that possess *bi*-, *tri*- or tetracyclic ring systems other than skeleton **1** and **2**. The side chain attached to C-11 in **1** or C-4 in **2** contains in some case double bonds, phenyl residues, or a methylenedioxyphenyl moiety. The substituents at C-6 in **1** or C-8 in **2** are usually a carboxylic acid, a phenyl ring or a methylenedioxyphenyl fragment.

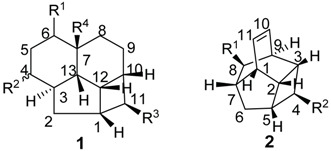


#### 2.1.1. Endiandric Acid Derivatives with an 13 Carbon Atoms Fused Tetracyclic Ring System (Table 1)

The first members of this class of compound, endiandric acid A and B (**3**–**4**) were isolated from the leaves of *Endiandra introrsa* [35,36,37,38,39]. Endiandric acid A (**3**) was also obtained from the leaves of other species such as *B. obtusifolia* and *B. oligandra* [40]. Endiandric acid B (**4**) was also isolated from *E. jonesii*, *E. baillonii* and *B. tooram* [40]. In addition to endiandric acid A (**1**), a new derivative, 3'',4''-methylenedioxy endiandric acid A (**5**) was obtained from *B. oligandra* after methylation of the extract and isolation of the non-natural methylated derivative **6** [40]. Endiandric acid H (**7**), a derivative with a hydroxyl group at C-4, was isolated from the stem of *Beilschmiedia fulva* [53,54]. Other endiandric acid analogues of this group with C_8_ alkyl side chain attached to the carbon C-11, named beilschmiedic acid A–E (**8**–**12**), in addition with beilschmiedic acid F (**13**) were isolated from the stem bark of *B. anacardioides* [17,19,20].

From the leaves of an unidentified *Beilschmiedia* species from Gabon, eight new beilschmiedic acid derivatives, named beilschmiedic acid H-O (**14**–**21**) were isolated using high-throughput natural products chemistry methods [48]. These compounds possess a phenylalkyl side chain at C-11, containing generally two *trans*-configured double bonds. A *cis* double bond, not reported previously in the side chain of endiandric acid derivatives was observed in beilschmiedic acid M (**18**) [48]. Beilschmiedic acid N (**20**) contains an unusual endoperoxide phenyl moiety that might have been formed during the process of isolation [48].

The phytochemical investigation of the root of *B. erythrophloia* resulted in the isolation of endiandric acid derivatives erythrophloins A–F (**22**–**27**) [41].

Endiandric acids with 13 carbon atoms fused tetracyclic ring system tsangibeilin A (**28**), tsangibeilin B (**29**), tsangibeilin C (**30**), tsangibeilin D (**31**) and the amide endiandramide A (**32)** have also been isolated from the roots of *B. tsangii* [22,23,52].

Four beilschmiedic acid derivatives with different oxidation states at C-4, cryptobeilic acids A–D (**33**–**36**), together with the known tsangibeilin B (**29**) were isolated from the bark of *B. cryptocaryoides* collected in Madagascar [52]. Other endiandric acid analogues, named ferrugineic acids A–J (**37**–**46**) were isolated from the leaves and flowers extracts of *B. ferruginea* with the help of ^1^H and ^13^C HSQC NMR screening of ethyl acetate extracts and fractions [24].

The investigation of the methanolic extract of the bark of *Endiandra kingiana* led to the isolation of further endiandric acid analogs kingianic acids F (**47**), G (**48**) and endiandric acid (**49**) [34].

**Table 1:** Substitution pattern of endiandric acid derivatives with skeleton **1**. 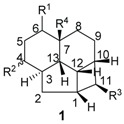

Compounds	R^1^	R^2^	R^3^	R^4^	Unsaturation	Sources	Ref.
Endiandric acid A (**3**)	Phenyl	H	CH_2_COOH	H	Δ^4,5^, Δ^8,9^	Leaves, *B. obstusifolia*, *B. tooram*, *E. introrsa*	[35,36,37,38,39,40]
Endiandric acid B (**4**)	Phenyl	H	CH_2_CH=CHCOOH	H	Δ^4,5^, Δ^8,9^	*Leaves, B. jonesii*, *E. introrsa, B. tooram*	[40]
3'',4''-methylenedioxy Endiandric acid A (**5**)	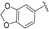	H	CH_2_COOH	H	Δ^4,5^, Δ^8,9^	Leaves, *B. oligandra*, stem bark, *B. manii,*	[40,44]
3'',4''-methylenedioxy Endiandric acid A methyl ester (**6**)	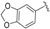	H	CH_2_COOMe	H	Δ^4,5^, Δ^8,9^	Synthesis, methylation of *B. oligandra* extract	[40]
Endiandric acid H (**7**)	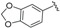	α-OH	CH_2_COOH	H	Δ^5,6^, Δ^8,9^	S*tem, B. fulva*	[53,54]
Beilschmiedic acid A (**8**)	COOH	β-OH	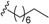	H	Δ^5,6^, Δ^8,9^	*Leave, Beilschmiedia* spp	[17,48]
Beilschmiedic acid B (**9**)	COOH	β-OH	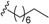	OH	Δ^5,6^, Δ^8,9^	*Leaves, Beilschmiedia* spp; Bark*, B. anacardioides*	[17]
Beilschmiedic acid C (**10**)	COOH	α-OH	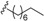	H	Δ^5,6^, Δ^8,9^	*Leaves, Beilschmiedia* spp; Bark*, B. anacardioides*	[17,48]
Beilschmiedic acid D (**11**)	COOH	H	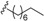	H	Δ^5,6^, Δ^8,9^	Bark*, B. anacardioides*	[20]
Beilschmiedic acid E (**12**)	COOH	H	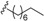	H	Δ^4,5^, Δ^8,9^	Bark*, B. anacardioides*	[20]
Beilschmiedic acid F (**13**)	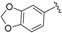	=O	CH_2_COOH	H	Δ^5,6^, Δ^8,9^	Bark*, B. anacardioides*	[19]
Beilschmiedic acid H (**14**)	COOH	α-OH	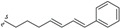	H	Δ^5,6^, Δ^8,9^	*Leave , Beilschmiedia* spp	[48]
Beilschmiedic acid I (**15**)	COOH	β-OH	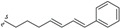	H	Δ^5,6^, Δ^8,9^	*Leaves, Beilschmiedia* spp	[48]
Beilschmiedic acid J (**16**)	COOH	H	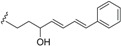	H	Δ^5,6^, Δ^8,9^	*Leaves, Beilschmiedia* spp	[48]
Beilschmiedic acid K (**17**)	COOH	α-OH	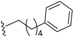	H	Δ^5,6^, Δ^8,9^	*Leaves, Beilschmiedia* spp	[48]
Beilschmiedic acid M (**18**)	COOH	α-OH	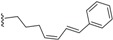	H	Δ^5,6^, Δ^8,9^	*Leaves, Beilschmiedia* spp	[48]
Beilschmiedic acid L (**19**)	COOH	α-OH	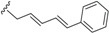	H	Δ^5,6^, Δ^8,9^	*Leaves, Beilschmiedia* spp	[48]
Beilschmiedic acid N (**20**)	COOH	α-OH	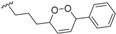	H	Δ^5,6^, Δ^8,9^	*Leaves, Beilschmiedia* spp	[48]
Beilschmiedic acid O (**21**)	COOH	α-OH	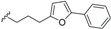	H	Δ^5,6^, Δ^8,9^	*Leaves, Beilschmiedia* spp	[48]
Erythrophloin A (**22**)	COOMe	H	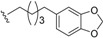	H	Δ^4,5^, Δ^8,9^	Roots, *B. erythrophloia*	[41]
Erythrophloin B (**23**)	COOMe	H	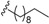	H	Δ^4,5^, Δ^8,9^	Roots, *B. erythrophloia*	[41]
Erythrophloin C (**24**)	COOMe	H	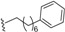	H	Δ^4,5^, Δ^8,9^	Roots, *B. erythrophloia*	[41]
Erythrophloin D (**25**)	COOMe	H	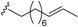	H	Δ^4,5^, Δ^8,9^	Roots, *B. erythrophloia*	[41]
Erythrophloin E (**26**)	COOH	H	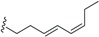	H	Δ^4,5^, Δ^8,9^	Roots, *B. erythrophloia*	[41]
Erythrophloin F (**27**)	COOH	H	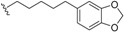	H	Δ^4,5^, Δ^8,9^	Roots, *B. erythrophloia*	[41]
Tsangibeilin A (**28**)	COOH	H	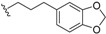	H	Δ^4,5^, Δ^8,9^	Roots, *B. tsangii*	[23]
Tsangibeilin B (**29**)	COOH	H	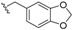	H	Δ^4,5^, Δ^8,9^	Roots, *B. tsangii*; Bark, *B. cryptocaryoides*	[34,41,52]
Tsangibeilin C (**30**)	COOH	=O	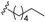	H	Δ^5,6^, Δ^8,9^	Roots, *B. tsangii*	[22]
Tsangibeilin D (**31**)	COOH	=O	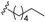	OH	Δ^5,6^, Δ^8,9^	Roots, *B. tsangii*	[22]
Endiandramide A (**32**)	CONHCH_2_-*i*pr	H	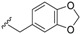	H	Δ^45^, Δ^8,9^	Roots, *B. tsangii*	[23]
Cryptobeilic acid A (**33**)	COOH	β-OH	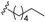	H	Δ^5,6^, Δ^8,9^	Bark, *B. cryptocaryoides*	[52]
Cryptobeilic acid B (**34**)	COOH	α-OH	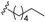	H	Δ^5,6^, Δ^8,9^	Bark, *B. cryptocaryoides*	[52]
Cryptobeilic acid C (**35**)	COOH	=O	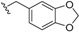	H	Δ^5,6^, Δ^8,9^	Bark, *B. cryptocaryoides*	[52]
Cryptobeilic acid D (**36**)	COOH	H	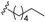	H	Δ^4,5^, Δ^8,9^	Bark, *B. cryptocaryoides*	[52]
Ferrugineic acid A (**37**)	COOH	H	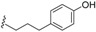	H	Δ^4,5^, Δ^8,9^	Leaves, flowers; *B. ferruginea*	[24]
Ferrugineic acid B (**38**)	COOH	H	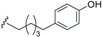	H	Δ^4,5^, Δ^8,9^	Leaves, flowers, *B. ferruginea*	[24]
Ferrugineic acid C (**39**)	COOH	H	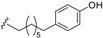	H	Δ^4,5^, Δ^8,9^	Leaves, flowers, *B. ferruginea*	[24]
Ferrugineic acid D (**40**)	COOH	H	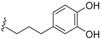	H	Δ^4,5^, Δ^8,9^	Leaves, flowers, *B. ferruginea*	[24]
Ferrugineic acid E (**41**)	COOH	H	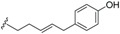	H	Δ^4,5^, Δ^8,9^	Leaves, flowers, *B. ferruginea*	[24]
Ferrugineic acid F (**42**)	COOH	α-OH	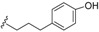	H	Δ^5,6^, Δ^8,9^	Leaves, flowers, *B. ferruginea*	[24]
Ferrugineic acid G (**43**)	COOH	β-OH	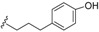	H	Δ^5,6^, Δ^8,9^	*B. ferruginea*	[24]
Ferrugineic acid H (**44**)	COOH	=O	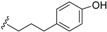	H	Δ^5,6^, Δ^8,9^	Leaves, flowers, *B. ferruginea*	[24]
Ferrugineic acid I (**45**)	COOH	α-OH	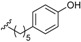	H	Δ^5,6^, Δ^8,9^	*B. ferruginea*	[24]
Ferrugineic acid J (**46**)	COOH	β-OH	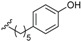	H	Δ^5,6^, Δ^8,9^	Leaves, flowers, *B. ferruginea*	[24]
Kingianic acid F (**47**)	COOH	H	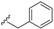	H	Δ^4,5^, Δ^8,9^	Bark, *E. kingiana*	[34]
Kingianic acid G (**48**)	COOH	α-OH	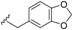	H	Δ^5,6^, Δ^8,9^	Bark, *E. kingiana*	[34]
Endiandric acid (**49**)	COOH	H	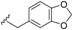	H	Δ^5,6^, Δ^8,9^	Bark, *E. kingiana*	[34]

#### 2.1.2. Endiandric Acid Derivatives with an 11 Carbon Atoms Fused Tetracylic Ring System (Table 2)

The first compound with this basic skeleton **2**, endiandric acid C (**50**), was isolated from the leaves of *Endiandra introrsa* [38,39,40]. This compound was also obtained from the leaves of other species such as *B. tooram*, *E. jonesii and E. baillonii* [40]. From the root of *B. erythrophloia*, endiandric acid I (**51**) and endiandric acid J (**52**) were isolated [49]. Further investigation of this species afforded a methylketone derivative named beicyclone A (**53**) [41]. The investigation of the roots of *B. tsangii*, afforded the new derivatives endiandric acids K (**54**), L (**55**), M (**56**), and endrindramide B (**57**) having an amide group at C-8 [23].

From the leaves of *B. ferruginea*, one endiandric acid analogue, ferrugineic acid K (**58**) also with skeleton **2**, was isolated. Other derivatives, kingianic acids A–E (**59**–**63**) with phenylalkyl side chains were isolated recently from the stem bark of *E. kingiana* [34].

**Table 2:** Structures of endiandric acid derivatives with skeleton **2**. 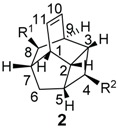

Compounds	R^1^	R^2^	Sources	Ref.
Endiandric acid C (**50**)	COOH	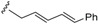	*B. tooram*, *B. oligandra*, *E. jonessi*, *E. introsa*	[38,40]
Endiandric acid I (**51**)	COOH	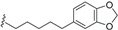	*Root*, *B. erythrophloia*	[49]
Endiandric acid J (**52**)	COOH	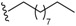	*Root*, *B. erythrophloia*	[49]
Beicyclone A (**53**)	COCH_3_	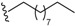	*Root*, *B. erythrophloia*	[41]
Endiandric acid K (**54**)	COOH	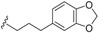	*Roots*, *B. tsangii*	[23]
Endiandric acid L (**55**)	CH=CHCOOH	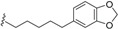	*Roots of B. tsangii*	[23]
Endiandric acid M (**56**)	CH=CH-COOH	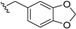	*Roots of B. tsangii, stem bark E. kingiana*	[23,34]
Endiandramide B (**57**)	CONHCH_2_*i*Pr	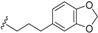	*B. tsangii*	[23]
Ferrugineic acid K (**58**)	CH=CH-COOH	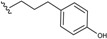	*B. ferruginea*	[24]
Kingianic acid A (**59**)	COOH	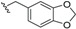	Stem bark, *E. kingiana*	[34]
Kingianic acid B (**60**)	COOH	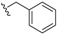	Stem bark, *E. kingiana*	[34]
Kingianic acid C (**61**)	COOH	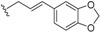	Stem bark, *E. kingiana*	[34]
Kingianic acid D (**62**)	COOH	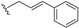	Stem bark, *E. kingiana*	[34]
Kingianic acid E (**63**)	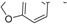	-CH_2_COOH	Stem bark, *E. kingiana*	[34]

#### 2.1.3. Other Endiandric Acid Derivatives

This group contains compounds that possess bi-, tri- or tetracyclic fused ring systems other than skeleton **1** and **2**. In this group are beilschmiedic acid G (**64**) having an aromatic ring and beilschmiedin (**65**) with a seven-membered cyclic ether group isolated from *B. anarcardiodes* [19,20]; tricyclotsangibeilin, an endiandric acid derivative with cyclododecane ring system (**66**) isolated from the roots of *B. tsangii* [22] and the bicyclic endiandric acids D (**67**), E (**68**), F (**69**) and G (**70**) isolated from *E. introrsa* [35,36,37,38,39,40].

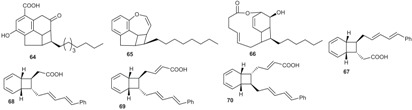


A total of 69 endiandric acid derivatives were isolated from 11 *Beilschmiedia* and four *Endiandra* plant species with the majority of these secondary metabolites having skeleton **1**.

#### 2.1.4. Biosynthesis of Endiandric Derivatives

**Scheme 1:**
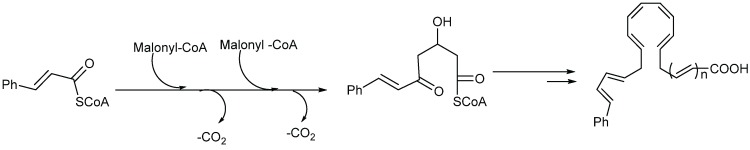
General biosynthesis scheme of polyketides.

Endiandric acids are polycyclic fatty acid derivatives with particular scaffolds isolated until date only in *Beilschmiedia* and *Endiandra* species of the Lauraceae family. They are products of electrocyclic ring closures of naturally occurring polyketides, resulting from both the shikimate and acetate pathways (Scheme 1).

**Scheme 2:**
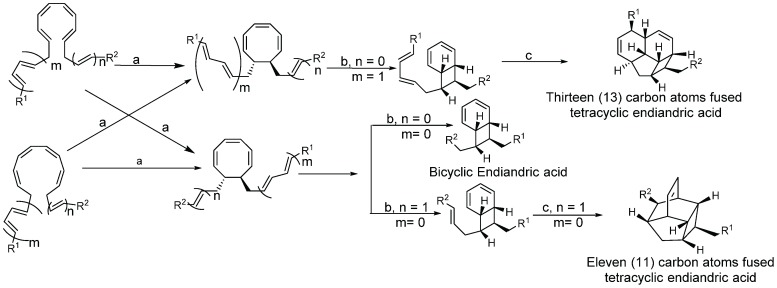
General biosynthesis scheme of endiandric acid skeleton from polyketides. a: Conrotatory 8π electron cyclization; b: Disrotatory 6π electron cyclization; c: Diels-Alder cyclization.

Their biosyntheses from the polyketide contain two consecutive non-enzymatic electrocyclic reactions, followed by an intramolecular Diels-Alder reaction [35,36,55]. As a result of the whole reaction sequence, an open-chain compound is converted into a tetracyclic compound. The starting product contains a conjugated tetraene system, as well as a conjugated diene system. Thus, it already displays the π electron systems required for the three pericyclic reactions; they are the two electrocyclizations and the Diels-Alder reaction [35,36,55] (Scheme 2).

**Scheme 3:**
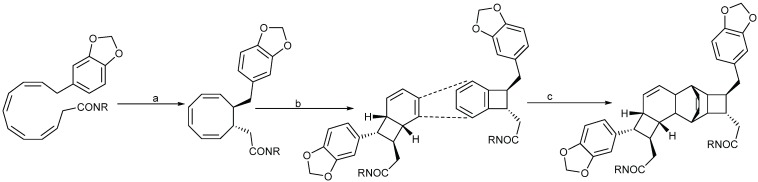
General biosynthesis scheme of kingianin derivatives. a: Conrotatory 8π electron cyclization; b: Disrotatory 6π electron cyclization; c: Diels-Alder cyclization.

Biogenesis of compounds of the kingianin family of natural products isolated from *E. kingiana* also involve a key Diels-Alder cycloaddition via a tandem 8π/6π electrocyclisation. In fact, an arylpolyene undergoes a conrotatory 8π *e* electrocyclization followed by a disrotatory 6π *e* electrocyclization of the formed cyclooctatriene. Radical cation formal Diels-Alder reaction between two bicyclo[4.2.0]octa-2,4-diene monomers led to unique and complex pentacyclic derivatives as shown in the Scheme 3 [42].

#### 2.1.5. Spectroscopic Characterization

The structures of endiandric acid derivatives have mainly been proposed on the basis of modern spectroscopic methods and sometimes X-ray diffraction analysis. The majority of these compounds possess a C_11_ or C_13_ tetracyclic ring system along with a number of double bonds and substituents that display characteristic spectroscopic properties.

##### 2.1.5.1. Mass Spectra

**Scheme 4:**
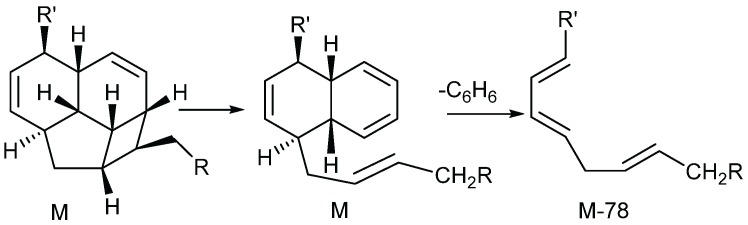
Fragmentation encountered in some endiandric acid [21].

**Scheme 5:**
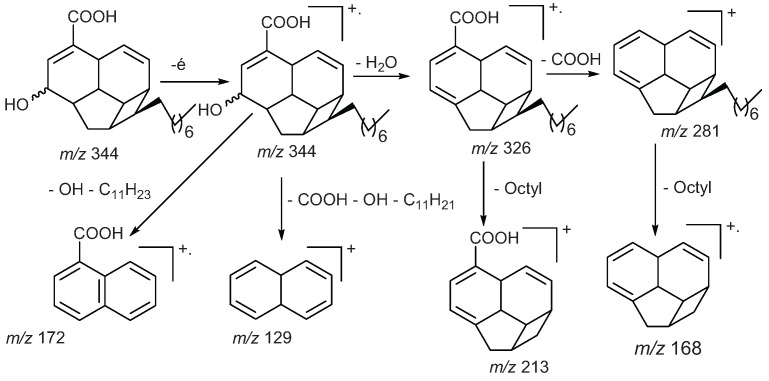
Fragmentation mechanism of beilschmiedic acid A and C, EI, 70 eV [17].

The mass spectrum of endiandric acid derivatives with C_13_ fused tetracyclic ring system having C^4^=C^5^ double bond generally exhibits an unusual fragmentation pattern where a strong M-78 ion is often observed, due to the loss of a benzene moiety (Scheme 4) [35,36]. Most of the EI spectra exhibited base peaks at *m/z* 172 or *m/z* 129 like those of beilschmiedic acid A and C (Scheme 5). Other fragments corresponding to cleavage of the side chain and the dehydration have also been observed (Scheme 5) [17,19,20].

##### 2.1.5.2. NMR Spectra

**Figure 1:**
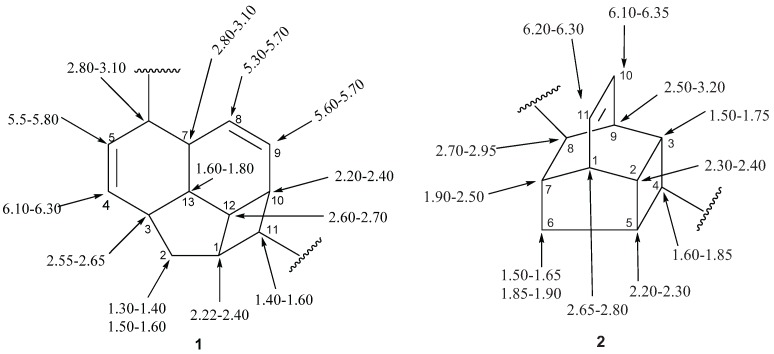
Range of ^1^H-NMR chemical shift of tetracyclic endiandric acid (skeleton **1** and **2**) [9,13,18,19,20,31,32,41,48,49,52].

The ^1^H-NMR spectra of endiandric acid derivatives are complex and structure elucidation has to rely on a combination of 1D- and 2D-NMR techniques. ^1^H-NMR spectra of derivatives with skeleton **1** having C^4^=C^5^ double bond showed, among other peaks, a broad singlet of the methine proton H-7 around 3.0 ppm, signals of one methylene protons [a doublet of triplet between δ 1.30–1.40 (H-2) and a doublet of doublet between 1.50–1.60 (H-2')], four *cis* olefinic protons at δ 6.10–6.30 (dt, H-4), 5.50–5.80 (dt, *J* = 9.7, 3.0 Hz, H-5), 5.30–5.70 (brd, *J* = 9.8 Hz, H-8), and 5.60–5.70 (dt, *J* = 10.2, 3.4 Hz, H-9). However, some signals are shifted when a double bond is present at C-5/C-6. In this case, a singlet of the olefinic proton H-5 is observed between δ 6.80–7.50 ppm. This proton resonates around 6.20 ppm when a ketone group is present at C-4. Endiandric acid derivatives with skeleton **2** exhibited, among other peaks, signals of methylene protons between δ 1.50–1.60 (H-6) and 1.80–1.90 (1H, H-6') and two *cis* olefinic protons between δ 6.10–6.30 (H-10) and 6.20–6.30 (H-11). Those of compounds with skeleton **1** or **2** bearing a methylenedioxyphenyl moiety exhibited in addition an ABX system of olefinic of three protons at δ 6.60–6.66 (1H, dd, *J* = 8.0, 1.6 Hz), 6.64–6.90 (1H, d, *J* = 1.6 Hz) and 6.72–7.20 (1H, d, *J* = 8.0 Hz) [17,19,20,22,23,24,41,44,48,49,52]. Figure 1 summarises the range of ^1^H-NMR data of endiandric derivatives.

**Figure 2:**
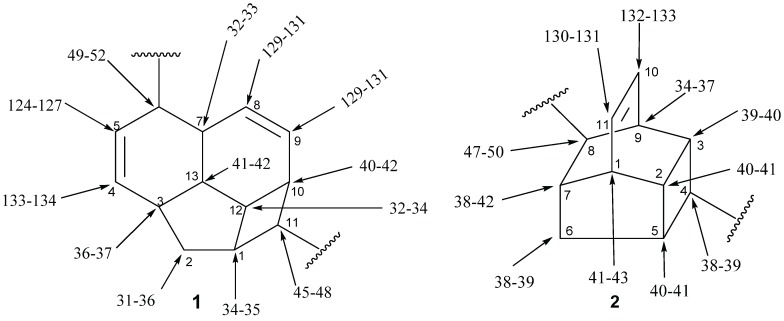
Range of ^13^C NMR chemical shift of the tetracyclic endiandric acid (skeleton **1** and **2**).

^13^C-NMR spectroscopy is very helpful in the structure elucidation of endiandric acid derivatives since chemical shift databases can be used to search for similar patterns. In addition, ^13^C-NMR chemical shifts tend to be more reproducible and are less influenced by solvent, temperature or pH [56,57]. The ^13^C-NMR spectrum of tetracyclic endiandric acids exhibits among other signals 11 or 12 methines and one to two methylenes for the basic skeletons [21,22,23,24,33,34,44]. The signals of methines (C-1, C-3, C-7, C-10, C-11, C-12, C-13) and the methylene C-2 in derivatives with skeleton **1** and those of methines (C-1, C-2, C-3, C-4, C-5, C-7, C-8, C-9) and the methylene C-6 in compounds with skeleton **2** are characteristic for the tetracyclic endiandric acids [9,14,21,34,44]. The chemical shift of the carboxy group at C-6 in compounds with skeleton **1** helps to confirm the location of the double bond in cycle D. In fact, when the double bond is C^5^=C^6^, the carbon of the carboxyl group appears at around 170 ppm. In case of C^4^=C^5^ double bond, it appears at between 175 and 181 ppm [9,13,14,18,19,20,30,31,44,48,52]. However, some signals are shifted when the double bond is C^5^=C^6^. In this case, the olefinic carbon C-5 appears at around 141 ppm in compound bearing a carboxylic group at C-6. This chemical carbon can shift to 145 ppm when hydroxyl is present at C-4 [9,13,18,19,20,31,32,48,52]. The chemical shift range (in ppm) of carbons of the tetracyclic endiandric skeletons are given in Figure 2.

### 2.2. Alkaloids and Amides

Plants of Lauraceae are rich sources of bioactive alkaloids [58,59,60,61,62,63,64,65]. The majority of alkaloids isolated from *Beilschmiedia* species possess aporphine skeletons (**71**, Table 3) or benzylisoquinoline (**72**) skeletons.

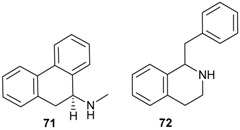


(+)-Predicentrine (**73**), which was first prepared by *O*-methylation of boldine (**74**), was isolated together with norpredicentrine (**75**) from the leaves of *B. podagrica*. Three aporphine alkaloids, isocorydine (**76**), glaucine (**77**), and (+)-*N*-methyllindcarpine (**78**) were obtained after subsequent investigation of the leaves of the same plant while laurelliptine (**79**) and isoboldine (**80**) were obtained from the bark [66,67]. Laurelliptine (**79**) was also obtained from the bark of *B. elliptica* together with the pale pink needles of isoboldine (**80**) [67,68]. Isoboldine (**80**) was identified as the major alkaloid from berries of *B. tawa* [46].

The phytochemical investigation of the leaves of *B. alloiophylla* has resulted in the isolation of the alkaloids 2-hydroxy-9-methoxyaporphine (**81**), laurotetanine (**82**), boldine (**74**), isoboldine (**80**), asimilobine (**83**) [29].

**Table 3:** Substitution pattern of aporphine alkaloids 73–86. 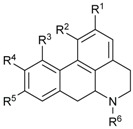

Compounds	R^1^	R^2^	R^3^	R^4^	R^5^	R^6^	Sources	Ref.
(+)-Predicentrine (**73**)	OH	OMe	H	OMe	OMe	Me	*B. podagrica*	[66]
Boldine (**74**)	OH	OMe	H	OMe	OH	Me	*B. alloiophylla*, *B. kunstleri*	[29]
Norpredicentrine (**75**)	OH	OMe	H	OMe	OMe	H	*B. podagrica*	[66]
(+)-Isocorydine (**76**)	OMe	OMe	OH	OMe	H	Me	*B. podagrica*	[66]
(+)-Glaucine (**77**)	OMe	OMe	H	OMe	OMe	Me	*B. podagrica*	[66]
(+)-*N*-methylindcarpine (**78**)	OH	OMe	OH	OMe	H	Me	*B. podagrica*	[66]
(+)-Laurelliptine (**79**)	OMe	OH	H	OMe	OH	H	*B. podagrica*	[67,68]
(+)-Isoboldine (**80**)	OMe	OH	H	OMe	OH	Me	*B. alloiophylla*, *B. tawa*	[29,46]
2-Hydroxy-9-methoxy aporphine (**81**)	OH	H	H	H	OMe	Me	*B. alloiophylla*	[29]
(+)-Laurotetanine (**82**)	OMe	OMe	H	OMe	OH	H	*B. alloiophylla*, *B. kunstleri*	[29,31]
(−)-Asimilobine (**83**)	OH	OMe	H	H	H	H	*B. alloiophylla*	[29]
(+)-Norboldine (**84**)	OH	OMe	H	OMe	OH	H	*B. kunstleri*	[31]
(+)-Cassithicine (**85**)	O-CH_2_-		H	OMe	OH	Me	*B. kunstleri*	[31]
Nornuciferine (**86**)	OMe	OMe	H	H	H	H	*B. kunstleri*	[31]

Tetracyclic alkaloids with morphine skeleton (+)-oreobeiline (**87**) and (+)-6-epioreobeiline (**88**) together with other aporphine alkaloids secoboldine (**89**), liriodenine (**90**) and (*S*)-3-methoxynordomesticine (**91**) were also isolated by Mollataghi and colleagues from *B. alloiophylla* [29]. Two of these alkaloids, (+)-oreobeiline (**87**) and (+)-6-epioreobeiline (**88**) were first isolated by Tillequin and colleagues from the wood of *B. oreophila* [47].

The benzylisoquinoline alkaloids O,O-dimethylannocherin A (**92**), (6,7-dimethoxy-4-methylisoquinolinyl)-(4'-methoxyphenyl)methanone (**93**), (6,7-dimethoxy-1-isoquinolinyl)-(4'-methoxyphenyl)methanone (**94**), (±)-*N*-norarmepavine (**95**), (*R*)-(−)-armepavine (**96**), *O*,*O*-dimethylcoclaurine (**97**) and *O*-methylvelucryptine (**98**) were isolated from the leaves of *B. brevipes* [45]. One compound of this class, (+)-*N*-dimethylphyllocryptine (**99**), was obtained from *B. alloiophylla* [29].

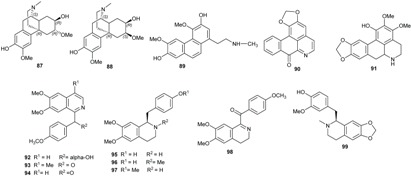


Dehatrine (**100**), an antimalarial bisbenzylisoquinoline alkaloid, was isolated in the frame of a bioguided investigation on antiplasmodial activity of the wood of the Indonesian medicinal plant *B. madang* [12].

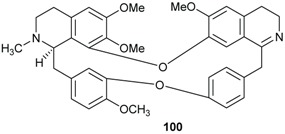


The phytochemical investigation of the leaves of *B. kunstleri* afforded several alkaloids: (+)-*N*-methylisococlaurine (**101**), (+)-cassythicine (**85**), (+)-laurotetanine (**82**), (+)-boldine (**74**), (−)-pallidine (**102**), (+)-nornuciferine (**86**), noratherosperminine (**103**), (+)-*N*-dimethylphyllocaryptine (**99**), (−)-isocaryachine (**104**), and the amide (−)-kunstleramide (**105**) [29,30]. Other amides, zanthonamide (**106**) and pipyahyine (**107**) have been isolated from the stem bark of *B. zenkeri* by Lenta and colleagues [18]. Additionally, a cyclostachine acid derivative obscurine (**108**) was isolated from the stem of *B. obscura* [28]. From the stem bark of *B. erythrophloia* three amides, *N*-*trans*-feryoltyramine (**109**), *N*-*trans*-feryoloctopamine (**110**) and beilschamide (**111**), were isolated [69].

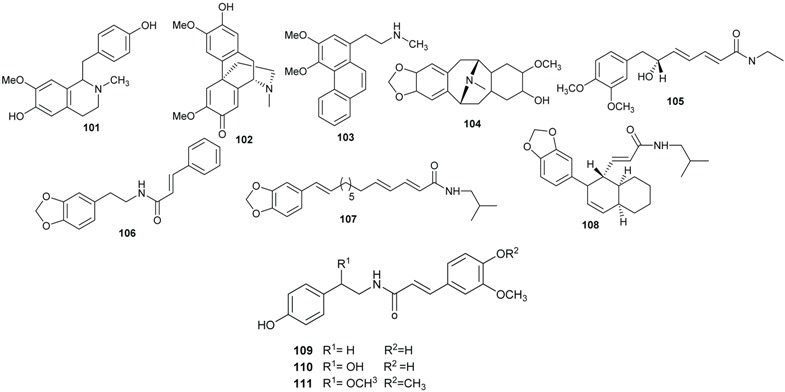


The chemical investigation of the bark of *E. kingiana* led to the isolation of a series of polyketides as a racemic mixtures, having each an amide function and named kingianins A–N (**112**–**125**). These amides possess an unusual pentacyclic carbon skeleton and was described for the first time in nature by Leverrier and coll. [42,43]. Seemingly, no alkaloids have been reported to date for the genus *Endiandra*.

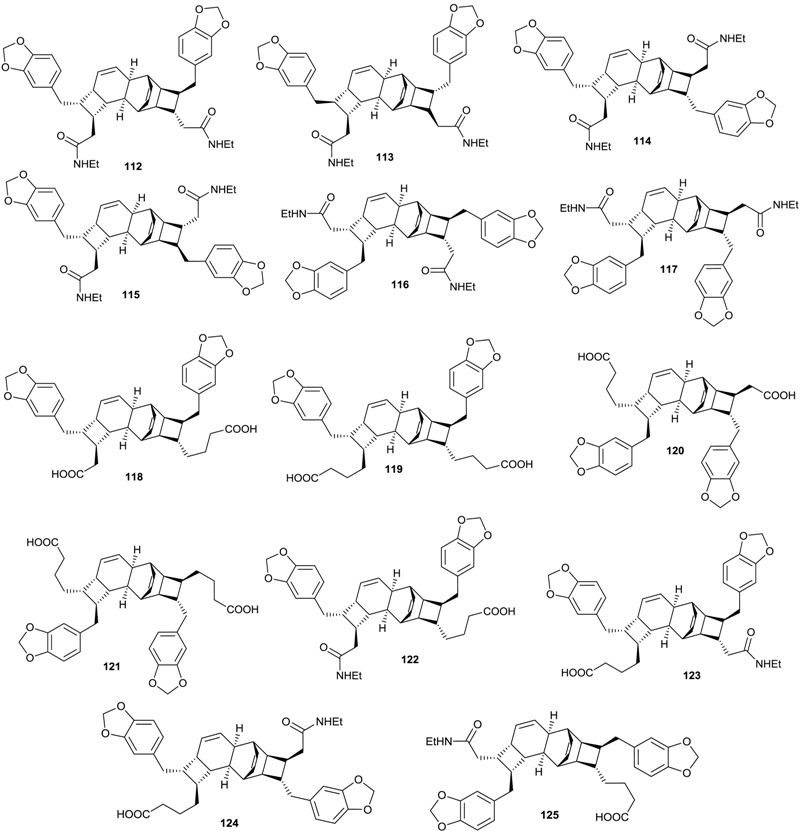


### 2.3. Lignans and Neolignans

Plants of the Lauraceae family are sources of bioactive lignans [70,71,72]. The majority of lignans and neolignans isolated from the genus *Beilschmiedia* were obtained from the species *B. tsangii*. The investigation of the roots, leaves and stem of this species afforded the lignans *ambo*-(7*R*,8*R*,7'*R*,8'*R*)-3',4'-methylenedioxy-3,4,5,5'-tetramethoxy-7,7'-epoxylignan (**126**), *ambo*-(7*R*,8*R*,7'*R*,8'*R*)-3,4,3',4'-dimethylenedioxy-5,5'-dimethoxy-7,7'-epoxylignan (**127**), beilschminol A (**128**), beilschminol B (**129**) together with other polymethylated derivatives 4α,5α-epoxybeilschmin A (**130**), 4α,5α-epoxybeilschmin B (**131**), beilschmin D (**132**), beilschmin A (**133**), beilschmin B (**134**), beilschmin C (**135**), *ambo-*(7*S*,8*S*,7'*R*,8'*R*)-3,3',4,4',5,5'-hexamethoxylignan (**136**), and three 1-phenylbutyl benzoates, tsangin A (**137**), B (**138**), and C (**139**) [22,23,25,26].

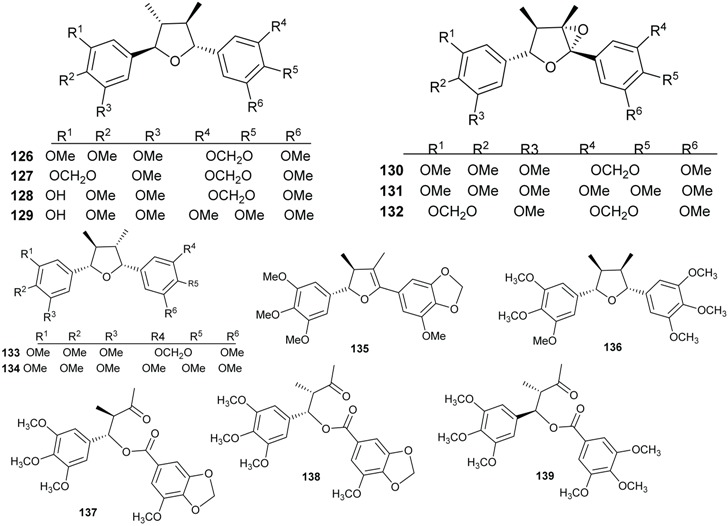


The investigation of the dichloromethane extract of the leaves of *B. kunstleri* yielded an antioxidant neolignan, (+)-kunstlerone (**140**) [30].

Magnolol (**141**), an antibacterial neolignan, was isolated from different *Endiandra* species specifically *E. xanthocarpa*, *B. volckii*, *E. bassaphila*, *E. leptodendron*, *E. monothyra* and *E. wolfii* [40]. From the extracts of the leaves of *E. xanthocarpa and E. palmerstonii*, sesamin (**142**), a lignan known to be a phytoestrogen, was isolated [40].

From the roots of *E. anthropophagorum* the cyclobutane lignans, endiandrin A (**143**) and endiandrin B (**144**), together with (−)-dihydroguaiaretic acid (**145**) and nectandrin B (**146**) were isolated. This type of lignan containing a cyclobutane moiety is rare in nature. Endiandrin A (**143**) and B (**144**) represent only the 23rd and 24th naturally occurring cyclobutane lignans [32,33].

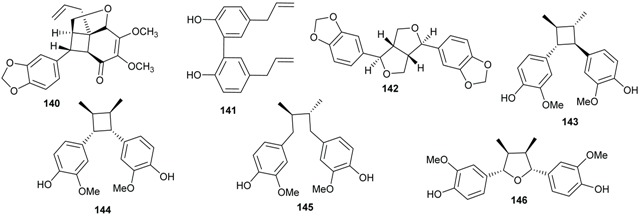


### 2.4. Flavonoids and Chalcones

Flavonoids have been isolated from many species of Lauraceae family [73]. However, this class of secondary metabolites has not been reported to date in *Endiandra*. They were obtained only from two species of *Beilschmiedia*: *B. miersii* and *B. zenkeri*. The rare quercetin-5-methyl ether known as azaleatin (**147**) was isolated from the leaves of *B. miersii* together with quercetin (**148**), quercetrin (**149**), isoquercetrin (**150**), afzeloside (**151**) and mikwelianin (**152**) [21].

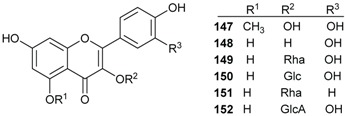


Five methoxylated flavonoid derivatives, (2*S*,4*R*)-5,6,7-trimethoxyflavan-4-ol (**153**), (2*S*,4*R*)-4,5,6,7-tetramethoxyflavan (**154**), beilschmieflavonoid A (**155**), beilschmieflavonoid B (**156**), and 5-hydroxy-7,8-dimethoxyflavanone (**157**), were isolated from the stem bark of *B. zenkeri* [18]. Beilschmieflavonoids A (**155**) and B (**156**) possess an unusual C_4_-O-C_4_'' linkage that has been found only in a biflavonoid isolated from *Tephrosia tepicana* (Leguminosae) [74]. Only two chalcones, 2',6'-dihydroxy-4-isoprenyloxy-3,4-(3''',3'''-dimethylpyrano)chalcone (**158**) and 4,2',6'-trihydroxy-3',4'-methylenedioxy-3-isopent-2-enylchalcone (**159**), were reported from the genus *Beilschmiedia* and precisely from the Brazilian species *Beilschmiedia tovarensis* [13]*.* No chalcone has been reported from the genus *Endiandra*.

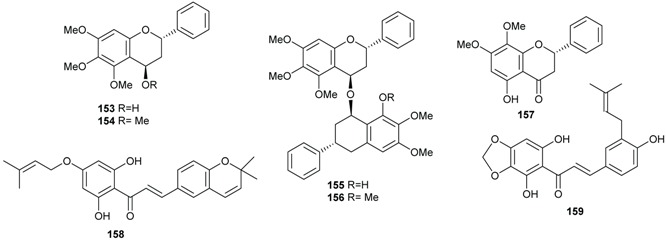


### 2.5. Benzene Derivatives

Benzene compounds have been isolated mostly from plants of the *Beilschmiedia* genus. Some of these compounds are benzaldehyde derivatives such as vanillin (**160**), *p*-hydroxybenzaldehyde (**161**), 3,4,5-trimethoxybenzaldehyde (**162**), isolated from the root and stem of *B. tsangii* [22,23,25]. Vanillin (**160**) was also isolated from the stem bark of *B. erythrophloia* [69]. Sarisan (**163**), an insect repelling allyl benzenoid, was isolated from the leaves of *B. miersii* [27,50]. The benzopyran oligandrol (**164**), was isolated from the bark of *B. oligandra* [40]. Its methylated derivative, oligandrol methyl ether (**165**), was obtained from the root of *B. erythrophloia* together with the benzenoids farnesylol (**166**) and α-tocopheryl quinone (**167**) [41,49]. Compound **167** was also isolated from the stem of *B. tsangii* and *B. erythrophloia* [25,69]. Another benzopyran derivative, α-tocopherol (**168**), was isolated from the roots of *B. tsangii*.

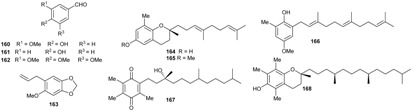


### 2.6. Terpenoids

Sesquiterpenes and triterpenoids are two classes of terpenoids mostly isolated from plants of *Beilschmiedia* and *Endiandra* genera.

The triterpenoids ursolic acid (**169**) and squalene (**170**) were isolated from the roots and leaves of *B. tsangii*, respectively [22,26]; lupeol (**171**) and 3-*O*-acetyl betulinic acid (**172**) were obtained from the roots of *B. erythrophloia*; betulinic acid (**173**) from the stem bark of *B. zenkeri* [18] and β-amyrone (**174**) from *B. alloiophylla* [29].

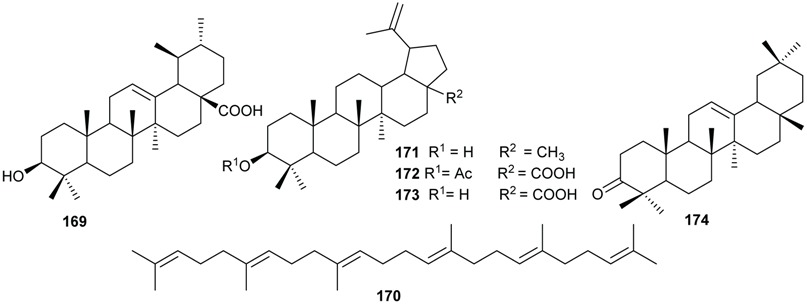


The skeletons of sesquiterpenes isolated from both genera are different. They are either bi-, tri-, or polycyclic with different degrees of oxidation. From the roots of *B. tsangii*, (+)-5-hydroxybarbatenal (**175**), (4*R*,5*R*)-4,5-dihydroxycaryophyll-8(13)-ene (**176**), octahydro-4-hydroxy-3α-methyl-7-methylene-α-(1-methylethyl)-1*H*-Indene-1-methanol (**177**), eudesm-4(15)-ene-1β,6α-diol (**178**), were isolated [22]. From its stem, 2,6,11-trimethyldodeca-2,6,10-triene (**179**) was obtained [25]. Suberosol B (**180**) was obtained from the roots of *B. erythrophloia* and (+)-α-curcumene (**181**) from the leaves of *E. xanthocarpa* [40]*.* The investigation of the leaves of another *Endiandra* species, *E. baillonii* also provided the sesquiterpenes ishwarane (**182**), α-copaene (**183**), cis/trans-calamanene (**184**), and (+)-α-curcumene (**181**) [40].

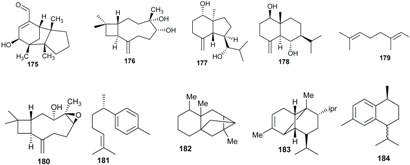


Sesquiterpenes and monoterpenes have been identified as the major constituents of the essential oils obtained from species of the genus *Beilschmiedia* (Table 4).

**Table 4:** Chemical composition of essential oils of *Beilschmiedia* species.

Species	Major Constituents
*B. miersii*	Leaf oil: Germacrene D (**185**, 24.8%), α-terpinene (**186**, 10%), γ-curcumene (**187**, 9.6%), 1-octen-3-yl acetate (**188**, 8.2%), (E)-β-ocimene (**189**, 6.4%) [51]
*B. tarairie*	Leaf oil: α-terpinene (**186**, 17.8%), β-pinene (**190**, 9.4%), germacrene D (**185**) [75]
*B. brenesii*	Leaf oil: germacrene D (**185**, 19.3, (*E*)-caryophyllene (**191**, 13.4%), 2-undecanone (**192**, 12.8%), α-copaene (**183**, 9.0), trans-2-hexanal (**193**, 8.8%) [76]
*B. costaricensis*	Leaf oil: α-bisabolol (**194**, 72.1%), *Cis* 2-hexenol (**195**, 5.2%), α-terpinene (**186**, 3.0%) [76]
*B. alloiophylla*	Leaf oil: germacrene D (**185**, (18.9), *cis*-β-ocimene (**196**, 18.8%), β-pinene (**190**, 3.0%), trans-β-ocimene (**189**, 9.3%), bicyclogermacrene (**197**, 9.1%) [77]
*B. talaranensis*	Leaf oil: germacrene D (**185**, 54.9%), β-caryophyllene (**191**, 14.8%), α-terpinene (**186**, 3.6%) [77]
*B. madang*	leaf oil: δ-cadinene (**198**, 17.0%), β-caryophyllene (**191**, 10.3%), α-cubebene (**199**, 11.3%), and α-cadinol (**200**, 5.8%) [78]; bark oil: δ-cadinene (**195**, 20.5%), β-caryophyllene (**191**, 6.7%), α-cubebene (**199**, 15.6%), and α-cadinol (**200**, 10.6%) [78]
*B."chancho chancho"*	Leaf oil: β-caryophyllene (**191**, 16.6%), bicyclogermacrene (**197**, 14.1%), β-pinene (**190**, 7.7%), germacrene D (**185**, 6.6%), δ-cadinene (**198**, 6.1%) [79]
*B. pendula*	leaf oil: β-pinene (**190**, 10.4%), β-caryophyllene (**191**, 8.6%), *c* (**201**, 7.9%) and bicyclogermacrene (**197**, 7.2%) [80]; branch oil: β-caryophyllene (**191**, 17.3%), β-selinene (**202**, 9.1%), bicyclogermacrene (**197**, 8.9%), α-cadinol (**200**, 5.8%) and spathulenol (**203**, 4.6%) [80]
*B. erythrophloia*	Leaf oil: β-caryophyllene (**191**, 22.6%), α-humulene (**204**, 21.9%), terpinen-4-ol (**205**, 5.3%), cis-β-ocimene (**196**, 5.1%), sabinene (**206**, 5.0%), limonene (**207**, 4.5%) [81]


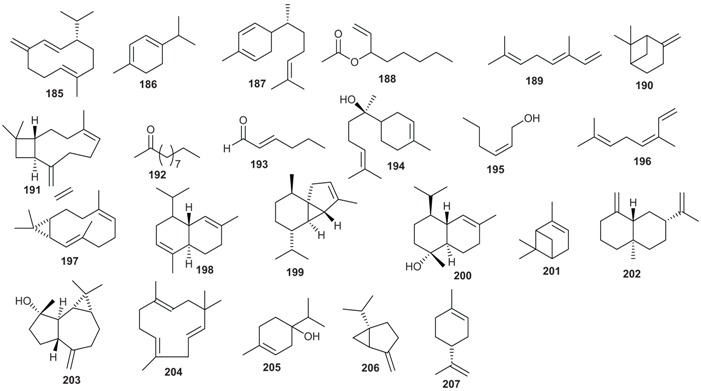


### 2.7. Cyanoglycosides

Chemotaxonomic studies indicated that terpenoids and alkaloids are common among plants of the Lauraceae family. In contrast, organic cyanides are very rare in Lauraceae and have only been reported from few species such as *Cinnamomum camphora*, *Litsea glutinosa* and *Nectandra megaptamica*. The investigation of 39 Australian Lauraceae species indicated that only *B. collina* was cyanogenic. In fact, from the methanol extract of *B. collina*, the first cyanogenic compound taxiphillin (**208**) was isolated from this genus [82].

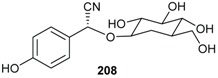


### 2.8. Other Metabolites

Steroids were isolated from extracts of many species from *Beilschmiedia*. Stigma-5-en-3-one (**209**), α-tocospiro B (**210**), 3β-hydroxystigma-5-en-7-one (**211**), stigmast-4-ene-3,6-dione (**212**), ergone (**213**), β-sitostenone (**214**), 6α-hydroxystigma-4-en-3-one (**215**) were isolated from the root of *B. tsangii* [25,26]. β-Sitosterol (**216**) was isolated from the stem of *B. tsangii* together with the pyrone derivatives α-tocospiro B (**217**) [25]. 6β-Hydroxystigma-4-en-3-one (**210**) and 3β-hydroxystigma-5-en-7-one (**211**) were also obtained from *B. erythrophloia* [41]. The fatty acid esters 2,3-dihydroxypropyl heptacosanoate (**218**), 1-(26-ferulyloxy hexacosanoyl)-glycerol (**219**) and 1-(26 hydroxyhexacosanoyl)-glycerol (**220**) were isolated from the stem of *B. obscura* together with 3β-acetylsitosterol (**221)** and β-sitosterol-3-*O*-d-glucopyranoside (**222**) [18]. β-Sitosterol (**216**) and β-sitosterol-3-*O-*d-glucopyranoside (**222**) were also isolated from *B. zenkeri* [18].

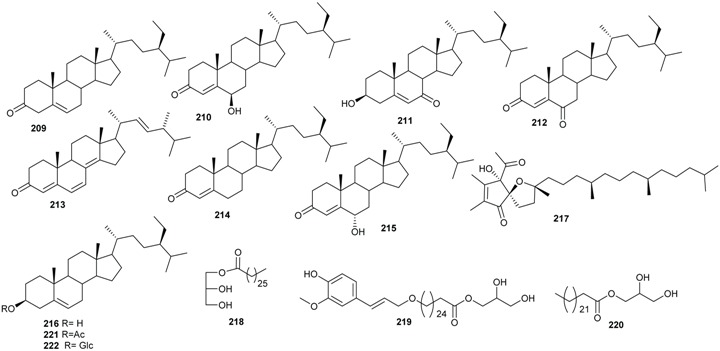


## 3. Biological Activities

Several interesting biological activities for the *Beilschmiedia* and *Endiandra* constituents have been reported, including antibacterial, anticancer, antifungal, antiinflammatory, antileishmanial, antiplasmodial and cytotoxic properties as well as α-glucosidase inhibiting activity.

### 3.1. Anticancer and Cytotoxic Activities

Cancer cells that avoid apoptosis continue to proliferate uncontrollably. Apoptosis is an ordered and orchestrated cellular process that occurs in physiological and pathological conditions. An understanding of the underlying mechanism of apoptosis is important as it plays a pivotal role in the pathogenesis of many diseases. Degenerative diseases are characterized by too much apoptosis, while in the case of cancer, too little apoptosis occurs. Thus, resisting apoptosis is a key process in cancer development and progression [83]. Targeting the antiapoptotic proteins such as those of the Bcl-2 family members (Bcl-2, Bcl-xL, Bcl-w, Mcl-1, and A1) is essential for cancer treatment or preventive drug discovery. In addition, it has been shown that most cancers depend on more than one antiapoptotic Bcl-2 member for survival. The discovery of new selective inhibitors of antiapoptotic proteins is thus important for the search for anticancer drugs [84,85,86,87].

Endiandric acids derivatives isolated from *Beilschmiedia* and *Endiandra* species were screened for Bcl-xL and Mcl-1 binding affinities (Table 5). Amongst the tested compounds, ferrugineic acid B (**38**) exhibited the best binding affinity for Mcl-1 (85% inhibition at 100 μM) while ferrugineic acid C (**39**) showed the highest binding affinity to Bcl-xL (93% inhibition at 100 μM). Two compounds, ferrugineic acids B (**38**) and C (**39**), exhibited significant binding affinities for both antiapoptotic proteins. Apart from tsangibeilin B (**29**) and ferrugineic acid J (**46**), the compounds that exhibited good binding affinity to Mcl-1 possess a C_13_ fused tetracyclic ring system with Δ^4,5^ and Δ^8,9^ double bonds. In the group of compounds with an C_11_ fused tetracylic ring system, only kingianic acid C (**61**) showed significant binding affinity to Mcl-1. No binding was detected from the compounds of this last fused tetracylic ring system for Bcl-xL. After the correlation between the structures and activities of compounds with C_13_ fused tetracyclic ring system, Appel and collaborators postulated that the length of the saturated carbon side chain, the β-oriented C-4 hydroxy group and the terminal 4-hydroxyphenyl ring, play a crucial role for Bcl-xL and Mcl-1 binding affinities [24,34].

**Table 5:** Binding affinities of some endiandric acid derivatives to antiapoptotic proteins Bcl-xL and Mcl-1.

Compound	Bcl-xL/Bak Binding Affinity		Mcl-1/Bid Binding Affinity	
	% at 100 μM	K_i_ μM	% at 100 μM	K_i_ μM
Tsangibeilin B (**29**)	26 ± 2.5	ND	81 ± 2.4	ND
Ferrugineic acid A (**37**)	22 ± 2	>100	0	14 ± 33
Ferrugineic acid B (**38**)	60 ± 6	19.2 ± 1.6	85 ±2	12.0 ±5.0
Ferrugineic acid C (**39**)	93 ± 3	12 ± 0.2	82 ± 2	13.0 ± 5.0
Ferrugineic acid D (**40**)	39 ± 3	>100	82 ± 2	5.2 ± 0.2
Ferrugineic acid E (**41**)	20 ± 1	ND	14.3 ± 3	ND
Ferrugineic acid F (**42**)	7 ± 1	ND	0	ND
Ferrugineic acid G (**43**)	17 ± 1	ND	3 ± 1	ND
Ferrugineic acid I (**45**)	35 ± 1	ND	7 ± 2	ND
Ferrugineic acid J (**46**)	58 ± 7	19.4 ± 3	81 ± 3	5.9 ± 0.5
Kingianic acid F (**47**)	22 ± 2.9	ND	80 ± 0.7	ND
Kingianic acid G (**48**)	19 ± 1.6	ND	47 ± 2.9	ND
Kingianic acid A (**54**)	21 ± 1.8	ND	36 ± 2.3	ND
Endiandric acid M (**56**)	10 ± 0.5	ND	39 ± 2.9	ND
Kingianic acid C (**61**)	25 ±1.7	ND	75 ± 1.1	ND
Kingianic acid E (**63**)	1 ± 0.8	ND	8 ± 5.5	ND
U-Bak (K_i_)		0.0012 ± 10^−3^		ND
U-Bid (K_i_)				0.016 ± 0.002
ABT-737 (K_i_)	57 ± 10 nM		47 ± 22 nM	

NT: Not tested; U-Bak and U-bid correspond to unlabeled peptides Bak and Bid, respectively.

The binding affinity of the racemic mixtures of kingianin A–N (**112**–**125**) isolated from *E. kingiana* was evaluated on Bcl-xL by competition against the fluorescently tagged BH3 domain of the protein Bak. Racemic mixtures of kingianins G–L (**118**–**123**) exhibited good binding affinity with kingianin G (**118**) exhibiting the best potency with a K_i_ value of 2 ± 0 μM (Table 6). The pure enantiomers of these active racemates obtained using chiral preparative HPLC were evaluated for their binding affinity. Taking into account the stereochemistry of the compounds, the binding affinity was significantly higher for the (−)-enantiomers compared to the (+)-enantiomers, as illustrated by the comparison of the K_i_ for (−) and (+)-kingianin derivatives (Table 6) [43].

**Table 6:** Bcl-xL binding affinity of compounds **112**–**125** (K_i_ in μM).

Compound	Bcl-xL K_i_		
	Racemic mixture	(−) Enantiomer	(+) Enantiomer
Kingianin A (**112**)	213 ± 83	60 ± 1.5	>300
Kingianin B (**113**)	>300		
Kingianin C (**114**)	>300		
Kingianin D (**115**)	>300		
Kingianin E (**116**)	>300		
Kingianin F (**117**)	231 ± 47		
Kingianin G (**118**)	2 ± 0	1.0 ± 0.2	5.0 ± 1.0
Kingianin H (**119**)	18 ± 7	4.0 ± 0.4	27.0 ± 0.6
Kingianin I (**120**)	18 ± 3	12.0 ± 1.1	16.0 ± 2.2
Kingianin J (**121**)	29 ± 6	9.0 ± 0.2	25.0 ± 3.2
Kingianin K (**122**)	80 ± 36	6.0 ± 0.2	112 ± 45
Kingianin L (**123**)	36 ± 11	4.0 ± 0.1	71.0 ± 10
Kingianin M (**124**)	236 ± 34		
Kingianin N (**125**)	177 ± 9		
Unlabeled Bak (BH3)	0.90 ± 0.27		

Endiandric acid analogues isolated from unidentified Gabonese *Beilschmiedia species* were screened for their cytotoxicity against NCI-H460 (human lung cancer cell lines); PC-3 (prostate adenocarcinoma cell lines), and M14 (amelanotic melanoma cell lines) using an MTT assay. All the isolated compounds were inactive against PC-3, and M14 cell lines. Beilschmiedic acids K (**17**), L (**19**), M (**18**), N (**20**) and A (**8**) exhibited moderate cytotoxicity against NCI-H460 human lung cancer cells with IC_50_ values of 5.5; 5.9; 4.4; 8.7; 19; 6.1 μM, respectively. This was the first report of the cytotoxicity of this class of secondary metabolites [48]. Subsequently, other endiandric acid derivatives kingianic acids A (**59**), C (**61**), E (**63**), F (**47**), G (**48**), endiandric acid M (**56**), and tsangibeilin B (**29**) isolated from *E. kingiana* were evaluated for their cytotoxicity activity against A549 (lung adenocarcinoma epithelial cell lines), HT29 (colorectal adenocarcinoma cell lines) and PC3 cell lines. As reported by Williams *et al.* [48], all compounds were inactive against prostate adenocarcinoma cancer cell lines. Kingianic acid A (**59**) showed weak activity against HT-29 and A549 cell lines with IC_50_ values of 35 ± 0.2 μM and 85.4 ± 0.2 μM, respectively. Kingianic acid E (**63**) showed moderate cytotoxic activity against A549 and HT-29 cell lines with IC_50_ values of 15.36 ± 0.19 μM and 17.10 ± 0.11 μM, respectively [34]. The other tested compounds showed very weak or were devoid of cytotoxic activity against the cell lines tested.

Ferrugineic acids A–J (**37**–**46**) and K (**58**), isolated from *B. ferruginea*, were screened for cytotoxicity against HCT-116 (Human colorectal carcinoma) and K562 (human leukemia) cancer cell lines. All these compounds were devoid of cytotoxicity on the two cancer cell lines tested at concentrations up to 50 µM [24].

The cytotoxicity activities of lignans and other constituents of the stem of *B. tsangii* were evaluated *in vitro* against P-388 and HT-29 cell lines. Beilschmin A (**133**), B (**134**), C (**135**), tsangin A (**137**), B (**138**), 2,6,11-trimethyldodeca-2,6,10-triene (**179**), α-tocopheryl quinone (**167**) and α-tocospiro B (**217**) were cytotoxic (IC_50_ below 4 µg/mL) against the P-388 cell lines. Tsangin A (**137**), B (**138**) and α-tocospiro B (**217**) were the most cytotoxic with IC_50_ values of 0.81 ± 0.009, 0.42 ± 0.03, and 0.83 ± 0.09 µg/mL, respectively, against the P-388 cell lines while 2,6,11-trimethyldodeca-2,6,10-triene (**179**) and α-tocospiro B (**217**) exhibited the best potency against the HT-29 cell line amongst the isolated with IC_50_ values of 2.2 ± 0.3 and 1.5 ± 0.2 µg/mL, respectively [25].

Endiandrin A (**143**), endiandrin B (**144**), (−)-dihydroguaiaretic acid (**145**) isolated from *E. anthropophagorum* and the synthesized derivative cinbalansan (**223**) were also evaluated for their cytotoxicity against A549 cell line. In high-content screening (HCS) assays, (−)-dihydroguaiaretic acid (**145**) was found to be the most potent compound, displaying cytotoxicity against the A549 cell line with an IC_50_ of 7.49 μM after 24 h incubation in both propidium iodide and Yo-PRO-1 assays. It effect was less pronounced in the mitotracker assay with IC_50_ of 31.2 μM. Endiandrin A (**143**), and B (**144**) were found to have moderate effects with an inhibition of 76% and 75% at 100 μM, respectively. Cinbalansan (**223**) was found to have much less effect with a maximum inhibition of 34% [32].

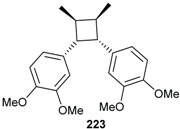


Alkaloids isolated from the leaves of *B. brevipes* exhibited cytotoxicity activity against P-388 murine leukemia cell lines. (6,7)-Dimethoxy-1-isoquinolinyl)-(4'-methoxyphenyl)methanone (**94**), *O*,*O*-dimethylcoclaurine (**98**), *O*-methylvelucryptine (**97**), (*R*)-(−)-armepavine (**96**) and (±)-*N*-norarmepavine (**95**) were active with IC_50_ values of 18.7, 6.5, 17.3, 42.2 and 44.5, respectively [45].

The stem bark and leaf extracts of *B. acuta* were evaluated against a panel of human cancer cell lines, including various multidrug-resistant phenotypes. The leaf extracts showed IC_50_ values below or around 30 mg/mL in 10 cell lines. Interestingly, among them were multidrug-resistant cell lines, e.g., P-glycoprotein overexpressing CEM/ADR5000, breast cancer resistance protein-transfected MDA-MB-231-BCRP, TP53 knockout cells (HCT116 p53^−/−^), and mutation-activated epidermal growth factor receptor-transfected U87MG.ΔEGFR cells [88].

(−)-Kunstleramide (**105**) a dienamide from *B. kunstleri* displayed cytotoxic effect in MTT assays against A375 (melanoma cell lines), A549, HT-29, PC-3 and WRL-68 (normal liver) cell lines with EC_50_ values of 64.65, 44.74, 55.94, 73.87 and 70.95 μg/mL, respectively [31]. Other amides *N*-*trans*-feryoloctopamine (**110**) and beilschamide (**111**) isolated from the stem of *B. erythrophloia* exhibited cytotoxic effects *in vitro*. They were active against CCRF-CEM (human lymphoblastic leukemia) cell line with IC_50_ values of 10.3 and 21.2 μg/mL, respectively [69].

The essential oil obtained from the leaf of *B. erythrophloia* exhibited cytotoxic activity against human OEC-M1 (oral squamous cancer), J5 (hepatocellular carcinoma), A549, HT-29, UACC-62 (melanoma) and K562 (leukemic) cell lines. The results showed that treatment with the essential oil for 48 h reduced the viability of OEC-M1 cells, J5 cells, A549 cells, HT-29 cells, UACC-62 cells, and K562 cells, with IC_50_ around 32.6, 48.6, 38.8, 18.9, 5.8, and 6.8 μg/mL, respectively [77].

### 3.2. Antimalaria Activity

Malaria remains one of the most notorious infectious diseases in the world. It constitutes a public health problem in more than 90 countries, inhabited by about 40% of the world’s population. The World Health Organisation estimates that there are 300–500 million malaria cases annually, causing 2–3 million deaths, mostly among children under five years old. In the last decades, resistance of *Plasmodium falciparum*, the causative agent of the most severe form of the disease, to several antimalarials, especially chloroquine and antifolates, became widely disseminated, while the cost of effective treatment is prohibitive for the large majority of the population in developing countries. For these reasons, new effective and affordable antimalarials are urgently needed [89,90,91]. In this perspective, extracts and some compounds isolated from *Beilschmiedia* species were screened for their antiplasmodial potency.

Cryptobeilic acids A–D (**33**–**36**) and tsangibeilin B (**29**) isolated from *B. cryptocaryoides* collected in Madagascar exhibited antiplasmodial activity *in vitro* against the chloroquine-sensitive strain of *P. falciparum* NF54 with IC_50_ values of 17.7, 5.35, 14.0, 10.8 and 8.2 µM, respectively. However, the cytotoxicity of these compounds against the L6 cell lines indicated low selectivity [52].

The antiplasmodial bioassay guided separation of the chemical constituents of the wood of the Indonesian medicinal plant *B. madang* led to the isolation of the bisbenzylisoquinoline dehatrine (**100**), that exhibited potent antiplasmodial activity against the chloroquine-resistant strain *P. falciparum* k1 with IC_50_ value of 0.17 μM, and which is comparable to that of the reference drug quinine against the same strain *in vitro* [12].

Pipyahyine (**107**), 5-hydroxy-7,8-dimethoxyflavanone (**157**), and betulinic acid (**174**) isolated from *B. zenkeri* exhibited antiplasmodial activity against the chloroquine-resistant strain of *P. falciparum* W2 with IC_50_ values of 3.7, 9.3 and 5.2 μM, respectively. Their activity were moderate compare to chloroquine (IC_50_ value of 0.13 μM), which was used as the positive control [18].

Lupeol (**171**), which showed *in vitro* inhibitory activity against the *P. falciparum* 3D7 strain with an IC_50_ value of 27.7 ± 0.5 μM, was shown to cause a transformation of the human erythrocyte shape toward that of stomatocytes [92].

### 3.3. Anti-Asthmatic and Other Anti-Inflammatory Activities

Asthma is a disease of the immune system, which is expressed for example as bronchial asthma in the form of acutely occurring, paroxysmal dyspnea with expiratory ventilation disability. Studies reported that persistent inflammation is central to the pathogenesis of asthma. So far, asthma therapy uses drugs which alleviate the symptoms but do not inhibit the mechanism responsible for the expression of inflammatory mediators such as the cytokines interleukin-4 (IL-4), interleukin-13 (IL-13) and interleukin-5 (IL-5) [93,94]. Endiandric acid H (**7**), obtained from the plant *Beilschmiedia fulva*, and its synthetic derivatives, known as c-maf, and NFAT inhibitors are used for producing a medicament, in particular for the treatment of allergic disorders, asthmatic disorders, inflammatory concomitant symptoms of asthma and/or of diseases which can be treated by inhibiting c-maf and NFAT [53,54].

The anti-inflammatory activities of extracts of *B. tsangii* have been studied. The methanol extract of the roots of *B. tsangii* showed potent inhibition of nitrogen monoxide (NO) production. Amongst the compounds isolated from this extract by bioassay guided separation, endiandric acid analogues endiandramide A (**32**) and B (**57**) with *N*-isobutylamide group exhibited potent iNOS inhibitory activities with IC_50_ values of 9.59 and 16.40 μM, respectively. Other isolates, tsangibeilin A (**26**), tsangibeilin B (**29**), endiandric acid K (**54**), endiandric acid M (**56**), endiandric acid L (**55**), and the lignans beilschminol A (**128**), beilschminol B (**129**), tsangin C (**139**) and tsangibeilin D (**31**), exhibited moderate anti-inflammatory activity with IC_50_ values in the range of 30–96 µM [22,23].

Synthetic glucocorticoids are widely used as drugs to treat inflammatory conditions such as rheumatoid arthritis or dermatitis and as adjunctive therapy for conditions such as autoimmune diseases. However, current glucocorticoid drugs act non-selectively, with the potential of long-term impairment of many physiological anabolic processes. Therefore, research aiming at the discovery of selective novel glucocorticoid receptor (GR) binders may provide new and improved drug therapies [95,96,97,98]. The bioguided fractionation of the dichloromethane extract of *Endiandra anthropophagorum* based on GR binding assay resulted in the isolation of the active lignans endiandin A (**143**), nectantin B (**146**) and (−)-dihydroguaiaretic acid (**145**) which displayed IC_50_ values of 0.9, 27 and 35 μM, respectively. The di-acetylated (**224**) and di-methylated (**225**) derivatives of endiandrin A also exhibited good activities with IC_50_ of 34 and 13 µM, respectively. From the structure–activity correlation, David and collaborators suggested that the constrained four-membered which has implications in the spatial arrangements of the substituents is important for the potent GR activity. In addition, increasing the steric bulk of the C-4/C-4' substituents in the cyclobutane series was shown to significantly reduce the activity [33].

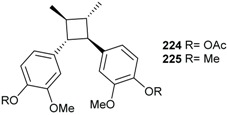


### 3.4. Antimicrobial Activity

Antibacterial activity of extracts and a number of endiandric acid derivatives and other constituents isolated from *Beilschmiedia* and *Endiandra* species have been studied.

Beilschmiedic acid A (**8**), B (**9**), and C (**10**), isolated from the stem bark of *B. anacardioides*, exhibited antibacterial activities against a wide range of microorganisms (*Bacillus subtillis*, *Micrococcus luteus*, *Streptococcus faecalis*) with minimum inhibitory concentrations (MICs) of 0.7–364 μM*.* Compound **10** was found to be the most active derivative against the three tested strains with MICs of 5.6, 0.7 and 22.7 μM, respectively. Compounds **9** (MIC value of 11.3 μM) and **10** (MIC value of 5.6 μM) were found to be more active than the reference drug ampicillin (MIC value of 89.5 µM) against *B. subtillis.* Compound **10** was also more active than the reference drug ampicillin (MIC value of 5.58 µM) against *M. luteus* [17].

Compound **8** and other endiandric acid derivatives beilschmiedic acids I–O (**15**–**21**), isolated from an unidentified Gabonese *Beilschmiedia* species, exhibited potent antibacterial activity against a clinical isolate of methicillin-resistant *S. aureus* (MRSA) with MIC values between 10 and 13 μg/mL [48].

Cryptobeilic acids A (**33**) and B (**34**) isolated from *B. cryptocaryoides* displayed antibacterial activity against *Escherichia coli* 6r3 with MIC values of 10 and 20 μg/mL, respectively. Their activity were moderate compared to that of the reference drug ampicillin (MIC value of 5 μg/mL) [52].

Endiandric acid erythrophloin C (**24**) with phenyl in the side chain isolated from *B. erythrophloia* exhibited antitubercular activity against *Mycobacterium tuberculosis* with MIC of 50 μg/mL [41].

In addition to endiandric acid derivatives, other constituents of *Beilschmiedia* and *Endiandra* genera have also exhibited antibacterial activity *in vitro* against some strains of bacteria. The amide pipyahyine (**107**) and beilschmieflavonoid B (**156**) isolated from the stem of *B. zenkeri* exhibited antibacterial activity *in vitro* against *Bacillus subtilis*, *P. agarici* and *S. minor* with MICs between 81.5–197.5 μM [18].

Magnolol (**141**), a neolignan isolated from different *Endiandra* species, showed strong antibacterial activities against both *Propionibacterium acnes* and *Propionibacterium granulosum*, which are acne-causing bacteria with the MIC value of 9 μg/mL [99].

Beilschmin A (**133**) and B (**134**), two lignans isolated from *B. tsangii*, exhibited potent antitubercular activities with MICs of 2.5 and 7.5 μg/mL, respectively. These compounds were more active than their corresponding epoxy-analogues, 4α,5α-epoxybeilschmin A (**130**) (IC_50_ of 30 µg/mL) and 4α,5α-epoxybeilschmin B (1**31** (IC_50_ of 40 μg/mL). As reported by Chen *et al.* [48], the epoxidation of the C4/C5 bond of compounds **133** and **134** considerably reduce their antibacterial activity against *M. tuberculosis*. Beilschmin A (**133**) with MICs of 2.5 μg/mL was most active than the reference compound, ethambutol (MIC of 6.2 µg/mL) [26]. The sesquiterpene suberosol B (**180**) isolated from *B. erythrophloia* also exhibited potent antitubercular activity against *M. tuberculosis* H37Rv *in vitro* with MIC of 28.9 µg/mL [41].

The methanol extract of the wood of *B. tovarensis* showed significant antibacterial activity results against *Staphylococcus aureus* and *Enterococcus faecalis* [13]. The methanol extract of the fruits of *B. obscura* showed antibacterial activity against multi-resistant drugs strains of *Escherichia coli*, *Enterobacter aerogenes*, *Klebsiella pneumoniae*, *Enterobacter cloacae*, *Pseudomonas aeruginosa*, and *Providencia stuartii* with MICs between 16–128 μg/mL [100].

Essential oils from the leaves and bark of *B. madang* showed moderate antibacterial activity towards *B. subtilis* and *S. aureus* with identical minimum inhibitory concentrations (MIC), 125 μg/mL. They also exhibited activity towards *E. faecalis* with MIC value of 250 μg/mL. Both oils were also found to be active against Gram-negative bacteria, *K. pneumoniae* with MIC value of 250 μg/mL [77].

The antifungal activities of extracts and the isolates from *Beilschmiedia* and *Endiandra* were also reported. The essential oils from the bark of *B. madang* showed strong antifungal activity towards *Aspergillus niger* and *A. fumigatus* with identical MIC values, 62.5 μg/mL [78].

The crude methanolic extract of *B. alloiophylla* was found to be active against *Candida albicans in vitro*. Alkaloids isolated from this extract boldine (**74**), 2-hydroxy-9-methoxyaporphine (**81**), laurotetanine (**82**), secoboldine (**89**), isoboldine (**80**), asimilobine (**83**), oreobeiline (**87**), 6-epioreobeiline (**88**), liriodenine (**90**), (*S*)-3-methoxynordomesticine (**91**) and the triterpenoid β-amyrone (**174**) exhibited good antifungal activity against the same strain with MICs in the range of 8–64 μg/mL [29]. Paulo and collaborators reported moderate antifungal activity for laurelliptine (**79**) and isoboldine (**80**) *in vitro* against *Tricophyton rubrum* and *Microsporum gypseun* [101].

### 3.5. Other Activities

Essential oil from the leaves of *Beilschmiedia madang* exhibited cholinesterase and tyrosinase inhibiting activities *in vitro* with inhibition of 55.2%, 60.4%, and 53.1% for acetyl-, butyrylcholinesterase and tyrosinase at 1000 μg/mL, respectively [78]. The crude methanolic extract of *B. alloiophylla* inhibited acetylcholinesterase and α-glucosidase *in vitro*. Four compounds from this extract, oreobeiline (**87**), 6-epioreobeiline (**88**), β-amyrone (**174**), and (*S*)-3-methoxynordomesticine (**91**), displayed moderate inhibitory activity against α-glucosidase with IC_50_ values of 8.0, 10.0, 20.0, and 10.0 μM, respectively. Other isolates from the same extract, 2-hydroxy-9-methoxyaporphine (**81**), laurotetanine (**82**) and liriodenine (**90**), displayed strong inhibitory activity against AchE with IC_50_ values of 2.0, 3.2 and 3.5 μM, comparable to that of the reference substance huperzine (IC_50_ values of 1.8 μM) [29].

The essential oils from the leaves of *B. tilaranensis* and *B. brenesii* exhibited enzyme inhibitory activities against cruzain, a potential therapeutic target for Chagas’ disease, a parasitic disease caused by *Trypanosoma cruzi* and that occurs mostly in South and Central American countries, with IC_50_ values of 23.6 μg/mL and 61.9 μg/mL, respectively [102,103,104].

The crude methanolic extract of *B. alloiophylla* was shown to exhibit antileishmanial activity *in vitro*. Compounds isolated from this extract, 2-hydroxy-9-methoxyaporphine (**81**), laurotetanine (**82**), liriodenine (**91**), boldine (**74**), secoboldine (**90**), isoboldine (**80**), asimilobine (**83**), oreobeiline (**87**), 6-epioreobeiline (**88**), β-amyrone (**174**), and (*S*)-3-methoxynordomesticine (**91**) exhibited moderate activity with IC_50_ values in the range of 10–50 μM [29].

The essential oils from *B. madang* exhibited weak DPPH radical scavenging activity (IC_50_ leaf oil, 263.9 μg/mL; bark oil, 212.0 μg/mL) compared to standard antioxidant, butylated hydroxytoluene (IC_50_ of 18.5 μg/mL) [78]. (−)-Kunstleramide (**105**) an dienamide from *B. kunstleri* exhibited very poor dose-dependent inhibition of DPPH (2,2-diphenyl-1-picrylhydrazyl) activity, with an IC_50_ value of 179.5 ± 4.4 μg/mL [31].

## 4. Conclusions

The genera *Beilschmiedia* and *Endiandra* include *ca.* 250 and *ca.* 125 species, respectively. Only 31 species of *Beilschmiedia* and 11 species of *Endiandra* have been investigated phytochemically, indicating that there are still many species that have received little or no attention. Secondary metabolites isolated from the two genera, can be classified as endiandric acid derivatives (30.9%), alkaloids and amides (23.7%), lignans and neolignans (9.5%), flavonoids and chalcones (5.8%) and others (30.21%: terpenoids, benzene derivatives, steroids, cyanoglycoside, fatty acids). Although being the majority of the isolates, the endiandric acid derivatives were not isolated from all species of *Beilschmiedia* or *Endiandra* investigated. This class of compounds and alkaloids is more widespread and the investigation of the remaining species could led to new derivatives. Bioassay on extracts and secondary metabolites of these species revealed pronounced biological properties, such as Bcl-xL and Mcl-1 binding affinity, α-glucosidase inhibiting activity, antimicrobial, anti-inflammatory, antiplasmodial, and cytotoxic properties. In addition to these bioactive secondary metabolites, essential oils from these species displayed good biological activities against a wide range of microorganisms and also enzyme inhibitory properties. The structural diversity of *Beilschmiedia* and *Endiandra* constituents and their interesting biological activities indicate that they are two potential sources of other new drugs such as that used in the treatment of asthma.
